# Nutritional risk, cold exposure, and stroke severity in older adults: an exploratory analysis of the “collapsed furnace” hypothesis

**DOI:** 10.3389/fpubh.2026.1838018

**Published:** 2026-08-26

**Authors:** Yumin Zhou, Yingying Huang, Qian Li, Dandan Lv, Yu Cao, Hao Tian, Weiwei Guo, Xia Bi

**Affiliations:** Shanghai University of Medicine and Health Sciences Affiliated Zhoupu Hospital, Shanghai, China

**Keywords:** CHARLS, cold stress, ecological study, malnutrition, oldest-old, sarcopenia, stroke, thermoregulation

## Abstract

**Objective:**

To evaluate the exploratory “Collapsed Furnace” hypothesis that nutritional depletion and sarcopenia-related frailty may alter responses to ambient cold in older adults with stroke.

**Methods:**

We integrated Global Burden of Disease (GBD) 2023 estimates for China (1990–2021), exploratory physiological data from the China Health and Retirement Longitudinal Study (CHARLS) 2015, and a retrospective clinical stroke cohort. The primary clinical analysis included patients aged ≥70 years; the full cohort aged ≥67 years was retained for sensitivity analysis. Nutritional risk was assessed using admission serum albumin as a nutritional-inflammatory marker, with the body mass index (BMI)-adjusted modified Geriatric Nutritional Risk Index (mGNRI) used as a sensitivity measure. Multivariable models examined nutritional risk, cold exposure, and their interaction in relation to admission National Institutes of Health Stroke Scale (NIHSS) score, adjusting for prespecified demographic, vascular, lifestyle, stroke-type, BMI, and C-reactive protein (CRP) covariates.

**Results:**

Sodium-attributable stroke burden peaked at ages 70–79 years, whereas cold-attributable burden increased in the oldest groups. In the GBD analysis, the malnutrition × low-temperature interaction was negative (*β* = −12.20, 95% confidence interval [CI], −18.52 to −5.88; *p* < 0.001). In the clinical cohort, nutritional risk was strongly associated with higher admission NIHSS scores. Cold exposure was associated with only a small additional NIHSS increase among patients with normal albumin and no measurable increase among those with low albumin; however, the interaction was not statistically significant. mGNRI- and CRP-based sensitivity analyses were directionally similar. CHARLS suggested different seasonal blood-pressure patterns by physiological status, but interpretation was limited by sparse cold-season interviews. Only 7 cold-exposed patients with low albumin aged ≥70 years and 4 CHARLS participants aged ≥70 years with cold-season interviews were available.

**Conclusion:**

Nutritional depletion was strongly associated with stroke severity, but its role in modifying cold-related vulnerability remains uncertain. Because the clinical interaction was not significant and mortality, pre-stroke albumin, individual cold exposure, and core temperature were unavailable, the proposed ceiling-like effect should be considered hypothesis-generating and requires prospective validation.

## 1 Introduction

Stroke is a leading cause of death and disability in China and poses a growing public health challenge as the population ages (1–3). Environmental exposures, including temperature variability, may further influence neurological risk (4–7). Stroke incidence and mortality commonly increase during winter. A widely proposed explanation is that cold exposure activates the sympathetic nervous system and peripheral vasoconstriction to reduce heat loss (8, 9). This response transiently raises systemic blood pressure (10) and, in people with arteriosclerotic vessels, may increase the likelihood of acute cerebrovascular events. Lower ambient temperatures have been associated with greater stroke morbidity and mortality in high-risk populations and may also destabilize vulnerable atherosclerotic plaques (11, 12).

Whether this conventional cold-stress model applies uniformly across the spectrum of frailty remains uncertain. A simple linear model assumes that progressively colder conditions produce progressively worse outcomes, but older adults vary substantially in nutritional status, muscle reserve, and autonomic capacity. In the oldest-old population (aged ≥85 years), severe physiological depletion may limit the ability to mount or sustain the usual vasoconstrictive and thermogenic responses. Consequently, the effect of ambient cold may be modified or overshadowed by low internal physiological reserve. This possibility has received limited attention in studies of stroke vulnerability.

We conceptualized this potential vulnerability as the ‘Collapsed Furnace’ hypothesis. Skeletal muscle is an important source of metabolic heat, in addition to its role in locomotion (13, 14), and adequate high-quality protein intake supports the maintenance of muscle mass and function (15). Malnutrition and sarcopenia, defined by age-related losses of muscle mass and function, may therefore reduce thermogenic and metabolic reserve in older adults (16–20). During cold exposure, people with limited muscle reserve may generate less heat or sustain compensatory responses less effectively. We evaluated this framework as an exploratory hypothesis rather than as an established biological mechanism.

Accordingly, we examined whether nutritional depletion was associated with altered vulnerability to cold exposure in older adults. The analysis integrated population-level estimates from the Global Burden of Disease (GBD) study, physiological data from the China Health and Retirement Longitudinal Study (CHARLS), and a retrospective clinical cohort. We tested an exploratory saturation or ceiling-like pattern while recognizing that observational data cannot establish the proposed mechanism. Because the cold-exposed low-albumin subgroup and the oldest CHARLS cold-season subgroup were small, the clinical and physiological analyses were intended to generate hypotheses rather than provide confirmation. Supplementary Figure S1 summarizes the complementary evidentiary roles of the three data sources and the safeguards applied to their interpretation.

## 2 Materials and methods

### 2.1 Data source I: population-level analysis (GBD 2023 database)

We used Global Burden of Disease (GBD) 2023 database exports from the Institute for Health Metrics and Evaluation (IHME) for China. The analysis was restricted to estimates for 1990–2021, the years available in the extracted files. The GBD framework provides standardized estimates of disease burden across 204 countries and territories (2, 3). We analyzed adults aged ≥70 years in 5-year age groups. The primary outcome was the rate of stroke disability-adjusted life years (DALYs) attributable to low temperature, expressed per 100,000 population. Exposure variables included the Summary Exposure Value (SEV) for low ambient temperature, high systolic blood pressure, and protein-energy malnutrition (PEM), defined by International Classification of Diseases, 10th Revision (ICD-10) codes E40–E46.

### 2.2 Operational definition: malnutrition as a proxy for sarcopenia

Because the GBD dataset does not contain direct measurements of skeletal muscle mass, we used the prevalence of protein-energy malnutrition (PEM) as an ecological proxy for compromised nutritional and metabolic reserve. Studies of geriatric and East Asian or Chinese older populations have reported associations among nutritional risk indicators, serum albumin, and sarcopenia-related measures, including the appendicular skeletal muscle mass index (ASMI) (18, 19, 21, 22). However, those populations are not identical to the GBD population structure and do not provide muscle measurements for the present analysis. PEM was therefore treated as an epidemiological proxy for compromised reserve, not as a direct measure of sarcopenia.

### 2.3 Data source II: retrospective clinical cohort

We conducted a single-center retrospective cohort study at Shanghai University of Medicine and Health Sciences Affiliated Zhoupu Hospital to examine the clinical relevance of the population-level findings. The Ethics Committee of the hospital approved the study (No. 2026-C-004) and waived the requirement for individual written informed consent because the analysis used anonymized, de-identified retrospective electronic medical records. All patient data were anonymized and de-identified before analysis in accordance with the Declaration of Helsinki.

We screened the electronic medical records of patients admitted to the Departments of Neurology and Rehabilitation Medicine with acute ischemic or hemorrhagic stroke between January 1, 2021, and December 31, 2024. The registry included patients aged ≥67 years who were admitted within 48 h of symptom onset and had complete laboratory data and admission National Institutes of Health Stroke Scale (NIHSS) scores. To improve comparability with the GBD age structure, the primary clinical analysis was restricted to patients aged ≥70 years; the full cohort aged ≥67 years was retained for sensitivity analysis. Patients with active malignancy, cachexia, or severe infection preceding stroke were excluded. Serum albumin was measured in the first routine venous blood sample obtained at admission after stroke onset. Because albumin is a negative acute-phase reactant, admission albumin was interpreted as a nutritional-inflammatory risk marker rather than a definitive measure of pre-stroke nutritional status. Patients were classified as having low-albumin nutritional risk (serum albumin <35 g/L) or normal albumin status (serum albumin ≥35 g/L).

#### 2.3.1 Meteorological data matching and threshold definition

Daily mean temperature data for 2021–2024 were obtained from the China Meteorological Data Service Center (CMDC). We used records from the Pudong National Meteorological Observation Station as the reference for the hospital catchment area. Missing observations (<0.1%) were imputed by linear interpolation. The primary exposure metric was the Lag 0–3 mean temperature, calculated from the day of stroke onset and the preceding 3 days. Cold exposure was defined as a Lag 0–3 temperature <5 °C. This area-level metric represented outdoor ambient conditions and did not capture indoor temperature, clothing, outdoor activity, or personal thermal protection.

#### 2.3.2 Stroke severity assessment and statistical handling

Stroke severity was quantified using the admission NIHSS score. For descriptive purposes, scores were categorized as minor (0–4), moderate (5–15), moderate to severe (16–20), or severe (21–42) (23, 24). In all multivariable linear regression and interaction analyses, NIHSS was modeled as a continuous outcome to preserve information and statistical power.

### 2.4 Statistical analysis

#### 2.4.1 Population-level analysis

We used a fixed-effects multivariable linear regression model to estimate the independent and interactive associations of malnutrition and cold exposure with the rate of stroke DALYs attributable to low temperature (per 100,000 population). Predictors included the low ambient temperature SEV, a risk-weighted measure ranging from 0 to 100, and malnutrition prevalence (%), used as a population-level proxy for metabolic reserve. Both continuous predictors were grand-mean centered before constructing their product term (malnutrition-centered × low-temperature-centered) to reduce structural multicollinearity. Fixed effects for 5-year age groups were included to account for age-related differences. We also fitted a reduced model without age-group fixed effects to assess the contribution of age structure to model fit. Model assumptions were evaluated using variance inflation factors (VIFs), residual normality checks, and residual-versus-fitted plots. Effect estimates are reported with 95% confidence intervals (CIs), and statistical significance was defined as a two-sided *p* value <0.05.

#### 2.4.2 Clinical validation analysis

For the retrospective clinical cohort, multivariable linear regression models were used to estimate associations with stroke severity, measured by the admission NIHSS score. The primary analysis included patients aged ≥70 years, and the full cohort aged ≥67 years was analyzed for sensitivity. Models were adjusted for age, sex, body mass index (BMI), hypertension, diabetes mellitus, atrial fibrillation, coronary artery disease, hyperlipidemia, smoking, alcohol consumption, stroke type (hemorrhagic vs. ischemic), and admission C-reactive protein (CRP). Nutritional risk × cold exposure product terms were used to test interaction. For a BMI-adjusted nutritional sensitivity analysis, we calculated a modified Geriatric Nutritional Risk Index (mGNRI): mGNRI = 1.489 × serum albumin (g/L) + 41.7 × min (BMI/22, 1). Nutritional risk was defined as mGNRI <98. Additional sensitivity analyses examined hot exposure, obesity (BMI ≥ 28 kg/m^2^), discharge modified Rankin Scale (mRS), and a low-inflammatory subgroup after exclusion of patients with CRP > 10 mg/L. The Supplementary Methods provide the exposure definitions and interaction formula.

### 2.5 Data source III: individual-level physiological context (CHARLS)

To provide individual-level physiological context, we used the 2015 wave of CHARLS, a nationally representative survey of Chinese residents aged ≥45 years (25). Participants were retained if they had complete data on age, sex, interview month, three systolic blood-pressure measurements, grip strength, height, and weight. Physiological frailty was defined as low grip strength (<28 kg for men or <18 kg for women, consistent with the Asian Working Group for Sarcopenia [AWGS] 2019 consensus and evidence from older Chinese cohorts (26–28)) or BMI < 18.5 kg/m^2^. Because few CHARLS 2015 interviews occurred during cold-season months, the analysis of systolic blood pressure (SBP) was considered exploratory physiological context rather than confirmatory mechanistic evidence.

The primary physiological outcome was mean SBP, calculated from three consecutive measurements (qa003, qa007, and qa011). Cold-season interviews were those conducted in January, February, November, or December. We report within-group SBP comparisons and the frailty × cold-season interaction while acknowledging the limited precision associated with the small cold-season sample. Supplementary Figure S2 shows the derivation of the CHARLS analytic samples.

### 2.6 Scenario-based predictive modeling

We constructed a counterfactual forecasting model for 2022–2035 to explore the potential population-level implications of changes in malnutrition prevalence. Trends in malnutrition prevalence and low-temperature exposure were projected within each age stratum using Holt’s linear exponential smoothing. A business-as-usual (BAU) scenario was compared with a hypothetical intervention scenario in which malnutrition prevalence decreased by 20% relative to the BAU baseline by 2030. These projections were scenario based and were not intended to estimate the effect of a tested intervention.

### 2.7 Comparison of data sources

The three data sources served complementary but non-equivalent evidentiary roles. Table 1 summarizes their designs, populations, analytic samples, exposures or markers, outcomes, and principal limitations.

**Table 1:** Comparison of the three data sources and their evidentiary roles.

Characteristic	GBD 2023	CHARLS 2015	Clinical cohort
Design	Population-level ecological analysis	Nationally representative longitudinal survey; cross-sectional 2015 analysis	Single-center retrospective hospital cohort
Population and period	China; age ≥70 years; 1990–2021	China; age ≥60 years, with an age ≥70 subset; 2015 wave	Shanghai stroke admissions; age ≥70 primary cohort and age ≥67 sensitivity cohort; 2021–2024
Analytic sample	Six 5-year age strata; 192 age-year observations	*N* = 7,633 aged ≥60 years; *N* = 2,739 aged ≥70 years	*N* = 300 aged ≥70 years; *N* = 350 aged ≥67 years
Key exposures or markers	Low-temperature SEV and PEM prevalence	Interview season, grip strength, and BMI-based frailty	Admission albumin, mGNRI, and Lag 0–3 outdoor temperature
Main outcome	Cold-attributable stroke DALY rate	Mean SBP	Admission NIHSS and discharge mRS
Evidentiary role	Population-level age-risk pattern and ecological interaction	Exploratory physiological context	Individual-level clinical association and sensitivity analyses
Principal limitations	Modeled ecological estimates; no direct muscle or individual exposure measurements	Only 21 cold-season interviews at age ≥60 years and 4 at age ≥70 years; interview season as exposure proxy	Post-stroke albumin; 7 cold-exposed patients with low albumin; survivor selection; no mortality or individual indoor exposure

BMI, body mass index; CHARLS, China Health and Retirement Longitudinal Study; DALY, disability-adjusted life year; GBD, Global Burden of Disease; mGNRI, modified Geriatric Nutritional Risk Index; mRS, modified Rankin Scale; NIHSS, National Institutes of Health Stroke Scale; PEM, protein-energy malnutrition; SBP, systolic blood pressure; SEV, Summary Exposure Value.

## 3 Results

### 3.1 Age-stratified risk profile

**Figure 1:**
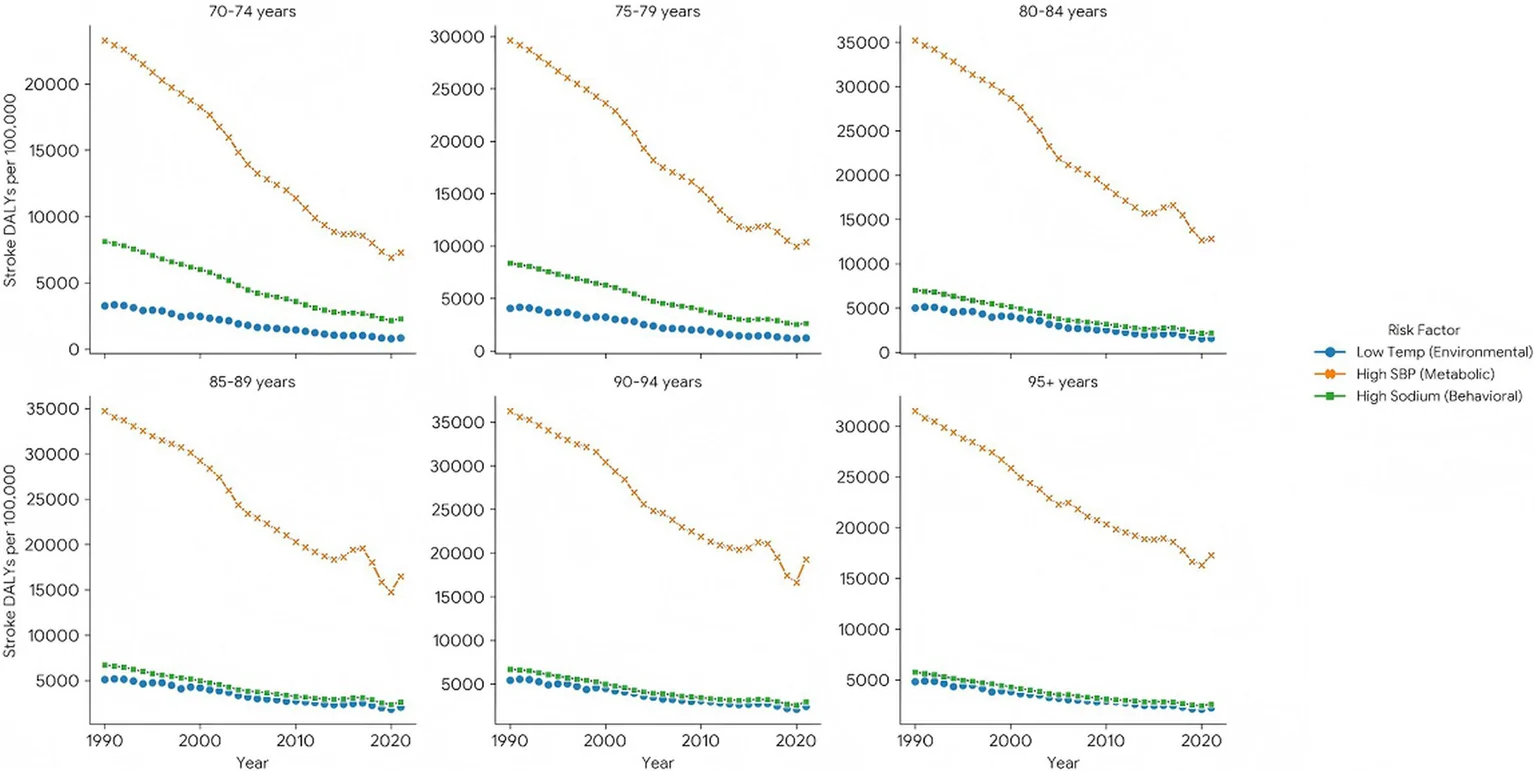
Temporal trends of stroke burden attributable to environmental, metabolic, and behavioral risk factors among older adults in China (1990–2021). The panels display the rate of stroke DALYs (per 100,000 population) stratified by 5-year age groups (70–74 to 95 + years). Blue line: Stroke DALYs attributable to low ambient temperature. Orange line: Stroke DALYs attributable to high systolic blood pressure (SBP). Green line: Stroke DALYs attributable to a diet high in sodium. Data source: Global Burden of Disease (GBD) 2023 database, restricted to 1990–2021 estimates. DALYs, disability-adjusted life years; SBP, systolic blood pressure.

The 2021 age-stratified estimates showed an age-related shift in the pattern of attributable stroke burden (Figure 1). High systolic blood pressure remained the largest and most consistent contributor across age groups, whereas the relative patterns of secondary risk factors differed with age.

Stroke burden attributable to a diet high in sodium peaked at ages 70–79 years and then plateaued or declined in the oldest groups (29). This pattern may indicate that the relative contribution of dietary sodium becomes less prominent at advanced ages, although selective survival and the increasing influence of other stressors are alternative explanations. In contrast, cold-attributable burden increased across the oldest age groups, particularly among adults aged ≥90 years. At ages ≥95 years, the increase in cold-attributable burden approached that of sodium-attributable burden. These descriptive patterns suggest that ambient temperature may become more relevant at advanced ages but do not establish an individual-level thermoregulatory mechanism.

### 3.2 Multivariable regression analysis and interaction pattern

**Figure 2:**
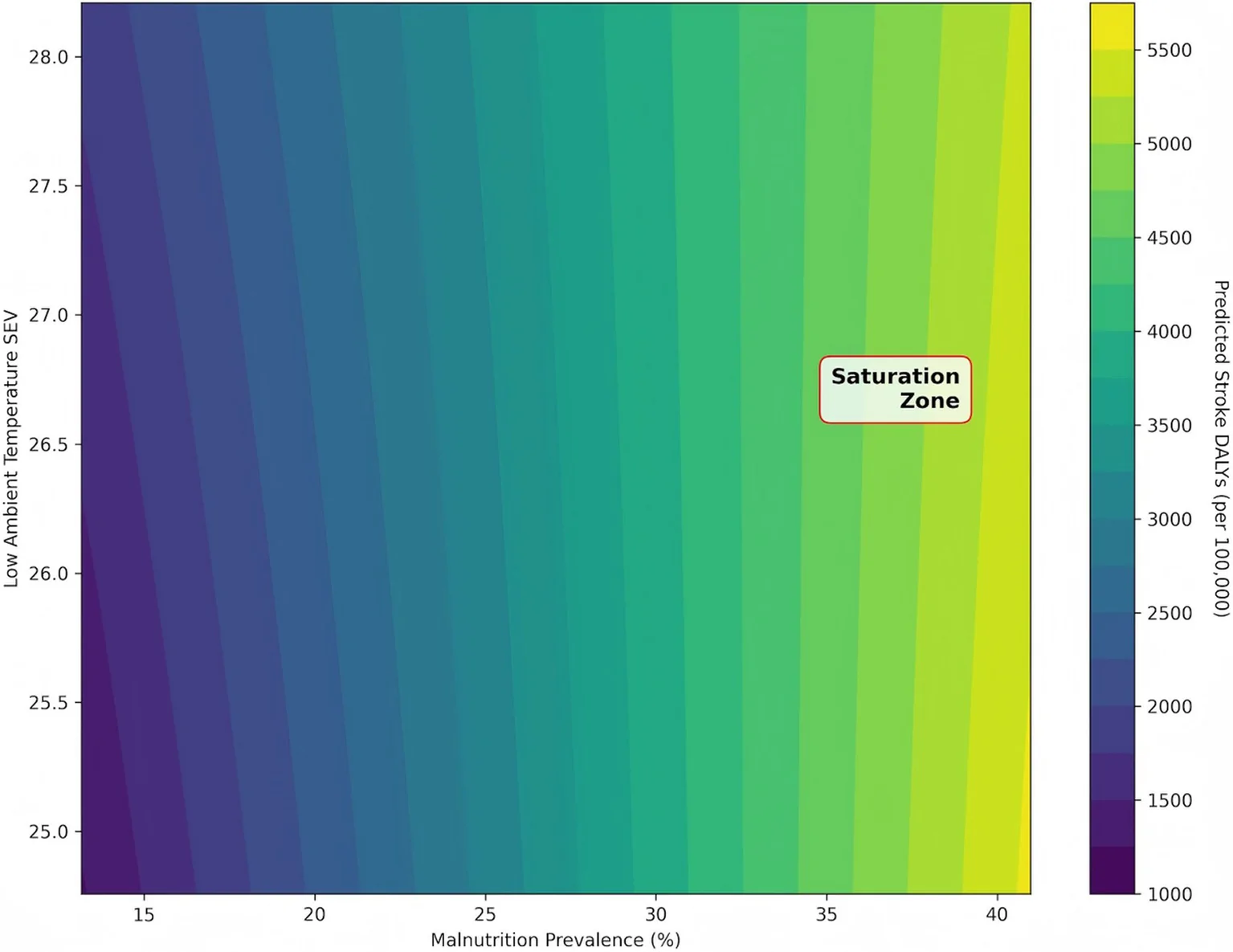
Contour plot of the interaction between malnutrition and low temperature on stroke burden. The heatmap displays the predicted cold-attributable stroke DALYs (per 100,000 population) as a function of low ambient temperature SEV and malnutrition prevalence (%), with age-group fixed effects held constant (averaged). X-axis: Malnutrition Prevalence (%). Y-axis: Low Ambient Temperature SEV (Summary Exposure Value, scale 0–100). Color gradient: Predicted Rate of Stroke DALYs. The annotated “saturation zone” in the upper-right region represents high malnutrition prevalence and high low-temperature SEV, where the modeled gradient flattens; this pattern corresponds to the interaction reported in Table 2. DALYs, disability-adjusted life years; SEV, summary exposure value.

In the fixed-effects model, malnutrition prevalence was positively associated with the rate of cold-attributable stroke DALYs (*β* = 151.53, 95% CI, 145.80 to 157.26; *p* < 0.001), as was the low ambient temperature SEV (*β* = 80.80, 95% CI, 35.36 to 126.25; *p* = 0.001). The interaction between the low-temperature SEV and malnutrition prevalence was negative (*β* = −12.20, 95% CI, −18.52 to −5.88; *p* < 0.001; Table 2), and Figure 2 shows the corresponding interaction surface. This ecological interaction was consistent with attenuation of the marginal cold-related burden at higher malnutrition prevalence but cannot establish individual-level causality. The full model with age-group fixed effects had *R*^2^ = 0.974 (adjusted *R*^2^ = 0.973), whereas the reduced model without age-group fixed effects had *R*^2^ = 0.938 (adjusted *R*^2^ = 0.937). Thus, age structure contributed to model fit, while the centered predictors and interaction remained informative in the reduced model.

**Table 2:** Multivariable fixed-effects regression analysis of stroke DALYs attributable to low temperature in older adults (GBD 2023 China data, 1990–2021 estimates).

Variables	Coefficient (*β*)	Standard Error (SE)	*t*-value	*p*-value	95% CI
Primary predictors					
Low ambient temperature (SEV)	80.80	22.96	3.52	0.001	35.36–126.25
Malnutrition prevalence (%)	151.53	2.89	52.35	< 0.001	145.80–157.26
Interaction term					
Malnutrition (%) × low temperature (SEV)	−12.20	3.19	−3.82	< 0.001	−18.52 – −5.88
Model diagnostics					
Observations (N)	192				
*R*^2^/adjusted *R*^2^ (full model)	0.974/0.973				
*F* statistic	873.0				
Fixed effects					
Age group stratification	Yes				

Associations of malnutrition prevalence, low-temperature exposure, and their interaction with cold-attributable stroke DALYs.

Model fit: full model with age-group fixed effects, *R*^2^ = 0.974 (adjusted *R*^2^ = 0.973); reduced model without age-group fixed effects, *R*^2^ = 0.938 (adjusted *R*^2^ = 0.937). Coefficients shown in this table are from the full model; the reduced model is reported only for comparison of model fit.

CI, confidence interval; SE, standard error; SEV, Summary Exposure Value; DALYs, disability-adjusted life years. The dependent variable is the rate of stroke DALYs attributable to low temperature (per 100,000 population). Continuous predictors (Low Temperature SEV and Malnutrition Prevalence) were grand-mean centered prior to interaction analysis. The full model includes fixed effects for 5-year age groups (70–95 + years).

### 3.3 Temporal trends and attribution patterns

**Figure 3:**
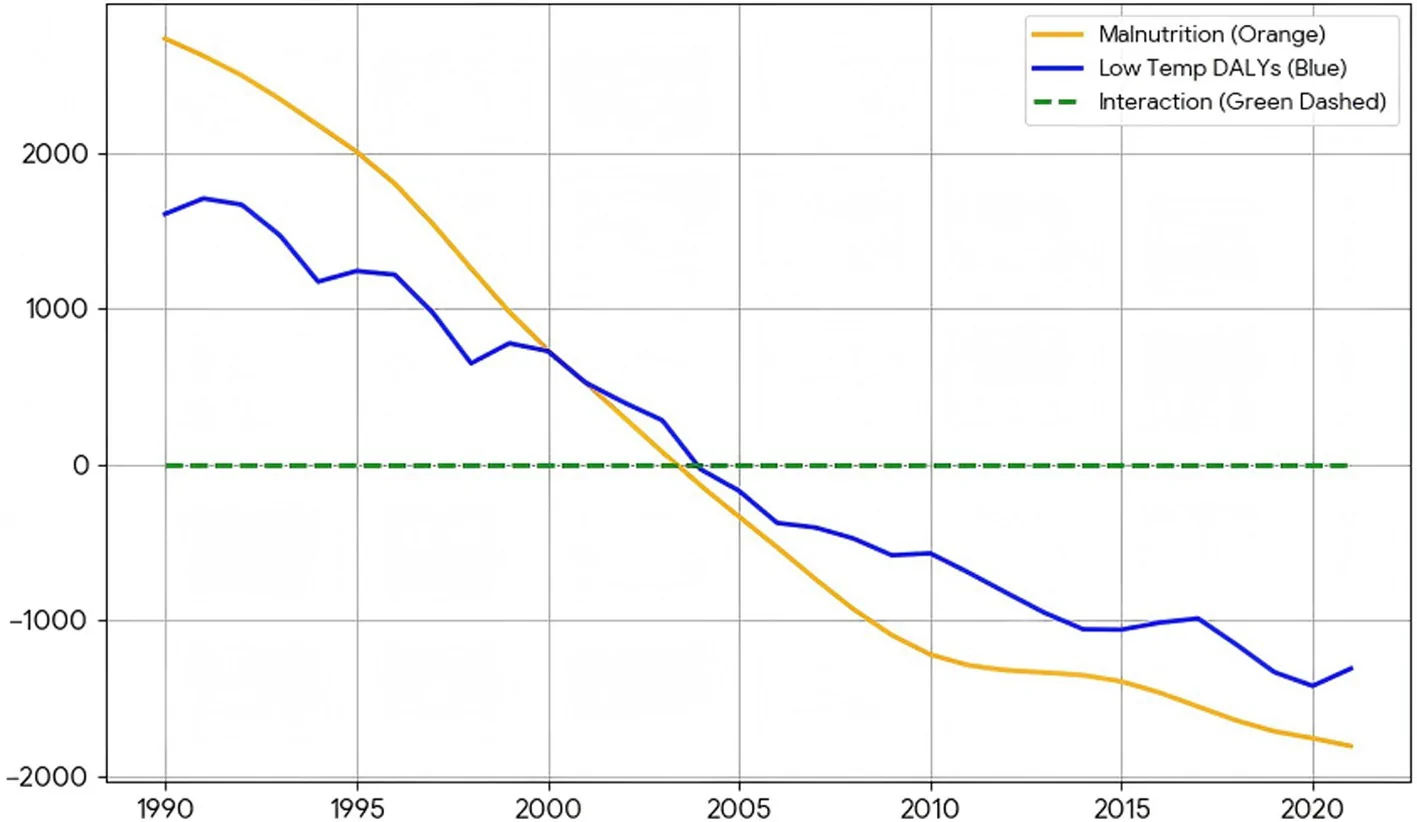
Centered temporal trajectories of risk factors and their interaction (1990-2021). Lines show mean-centered temporal trajectories derived from the Global Burden of Disease (GBD) 2023 database, restricted to 1990-2021 estimates. Orange line: trend of stroke DALYs attributable to malnutrition. Blue line: trend of stroke DALYs attributable to low temperature. Green dashed line: statistical interaction between malnutrition and low temperature. DALYs, disability-adjusted life years.

From 1990 to 2021, the burden attributable to malnutrition declined, whereas the rate of cold-attributable stroke DALYs remained high and fluctuated over time (Figure 3). The contrasting trajectories indicate that declining malnutrition prevalence did not coincide with a comparable reduction in cold-attributable burden among older adults.

Further visual decomposition (Figure 3C, Green Dashed Line) showed that the estimated interaction term remained close to baseline over time. This pattern should be interpreted descriptively because temporal stability of an ecological interaction term cannot establish a persistent biological mechanism or individual-level causality.

### 3.4 Physiological context (CHARLS): exploratory SBP analysis

**Figure 4:**
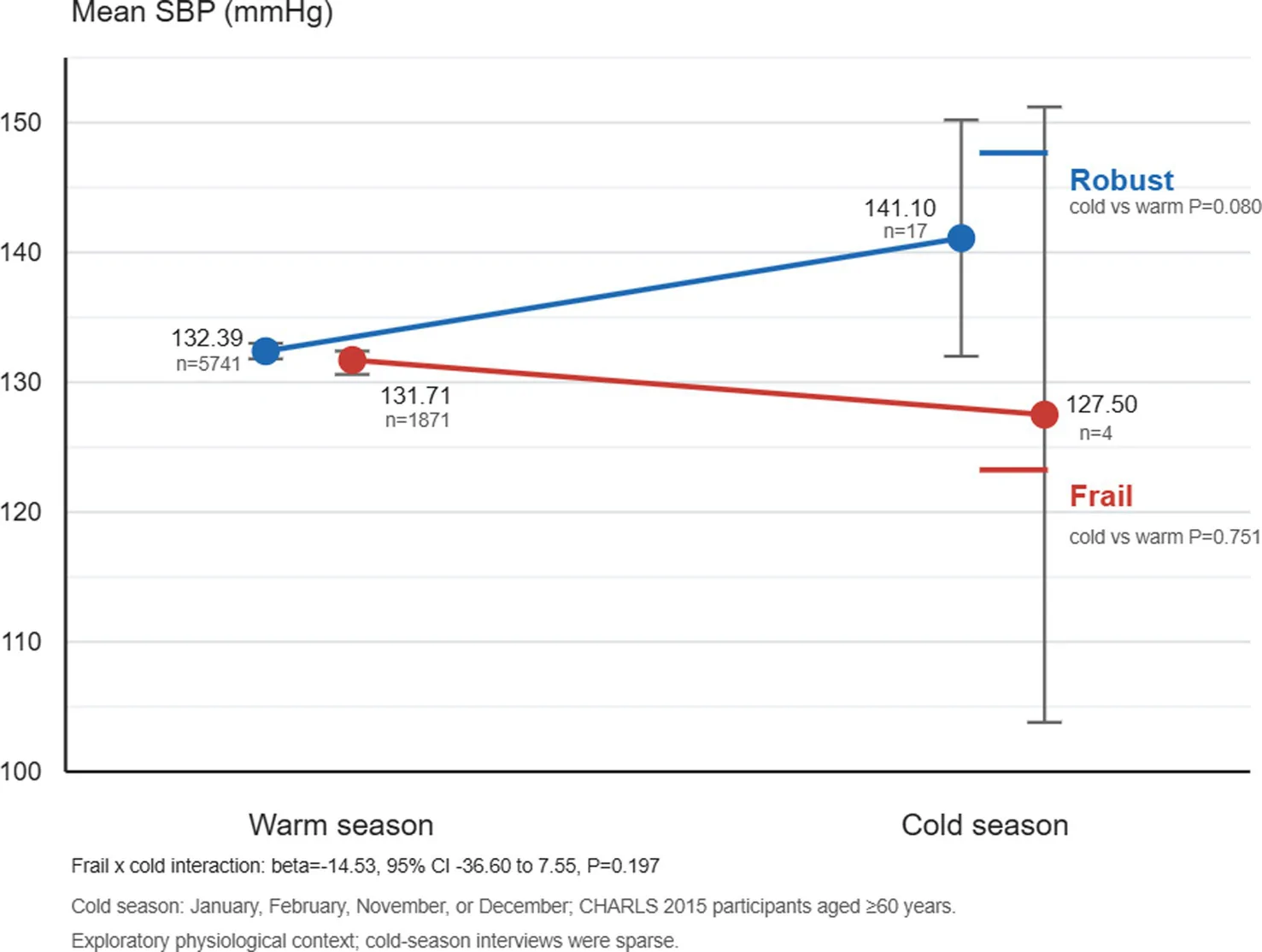
Exploratory seasonal systolic blood pressure (SBP) analysis in the China Health and Retirement Longitudinal Study (CHARLS) 2015, stratified by physiological status. SBP was calculated as the mean of three consecutive measurements. Robust status was defined by normal grip strength and body mass index (BMI); frailty was defined by low grip strength or BMI < 18.5 kg/m^2^. Cold season was defined as interviews in January, February, November, or December; warm season included all other interview months. Cold-season interviews were sparse in CHARLS 2015. Among participants aged ≥60 years with complete data, only 21 were interviewed during cold-season months; among participants aged ≥70 years, only 4 cold-season interviews were available. Within-group comparisons showed that robust participants had a higher mean SBP in cold-season than warm-season interviews (141.10 ± 19.19 vs. 132.39 ± 20.36 mmHg; *p* = 0.080), whereas frail participants did not (127.50 ± 24.18 vs. 131.71 ± 22.50 mmHg; *p* = 0.751). The adjusted frailty × cold-season interaction was negative but not statistically significant (*β* = −14.53, 95% CI, −36.60 to 7.55; *p* = 0.197). This analysis should therefore be interpreted as exploratory physiological context rather than confirmatory mechanistic evidence. CI, confidence interval.

Among CHARLS participants aged ≥60 years with complete data, only 21 of 7,633 were interviewed during cold-season months; among those aged ≥70 years, only 4 cold-season interviews were available. Based on the mean of three measurements, robust participants had a higher mean SBP in cold-season than warm-season interviews (141.10 ± 19.19 vs. 132.39 ± 20.36 mmHg; *p* = 0.080), whereas frail participants did not (127.50 ± 24.18 vs. 131.71 ± 22.50 mmHg; *p* = 0.751; Figure 4). The adjusted frailty × cold-season interaction was negative but not statistically significant (*β* = −14.53, 95% CI, −36.60 to 7.55; *p* = 0.197). These results provide exploratory physiological context but are imprecise because of the sparse cold-season sample.

### 3.5 Scenario-based projection

**Figure 5:**
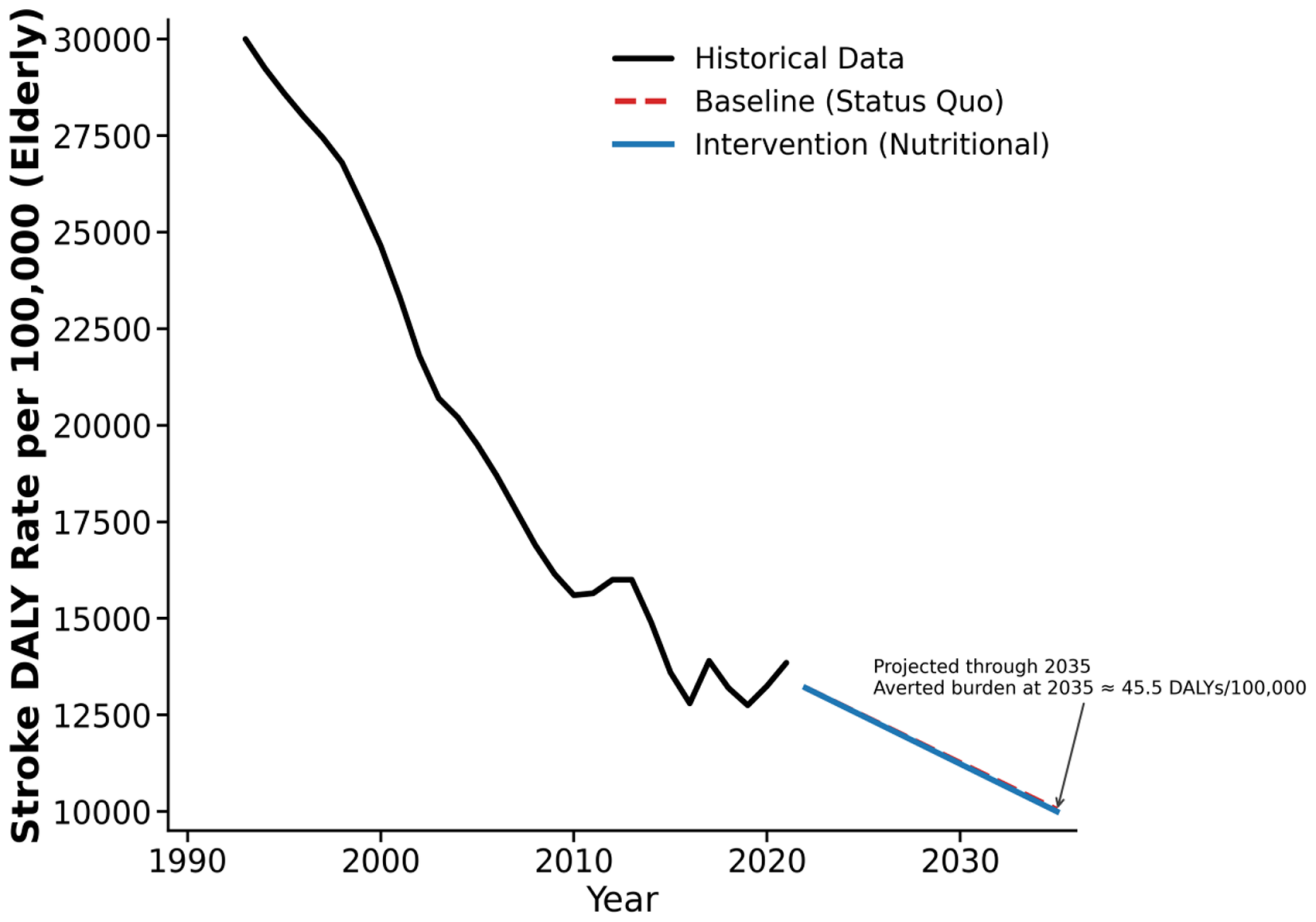
Scenario-based projected trajectories of cold-attributable stroke DALYs among older adults in China, 2022-2035. This is a scenario-based predictive projection and should not be interpreted as evidence of real-world intervention efficacy. Historical data (black line): observed trend from 1990 to 2021. Baseline scenario (red dashed line): projected burden under continuation of current trends in malnutrition and cold exposure (business-as-usual [BAU]). Intervention scenario (blue solid line): projected burden assuming a 20% reduction in malnutrition prevalence relative to the baseline by 2030. The annotated difference at 2035 represents the estimated averted burden between the baseline and intervention scenarios (approximately 45.5 DALYs per 100,000). DALYs, disability-adjusted life years.

The scenario-based model projected cold-attributable stroke DALY rates for 2022–2035 from forecast trends in malnutrition prevalence and low-temperature exposure (Figure 5). Predictor trajectories were estimated using damped-trend Holt’s linear exponential smoothing. Under the hypothetical intervention scenario, in which malnutrition prevalence decreased by 20% relative to the BAU baseline by 2030, the model estimated approximately 45.5 fewer DALYs per 100,000 population in 2035, a relative difference of about 0.3%. This estimate illustrates the direction and scale of a counterfactual scenario; it is not evidence of the effectiveness of nutritional intervention or a forecast of observed policy impact.

### 3.6 Clinical validation: nutritional risk, cold exposure, and stroke severity

#### 3.6.1 Baseline impact of nutritional status

The primary clinical cohort included 300 patients aged ≥70 years: 87 had serum albumin <35 g/L and 213 had serum albumin ≥35 g/L. Patients with low albumin had higher admission NIHSS scores than those with normal albumin (16.3 ± 3.3 vs. 8.2 ± 4.8; *p* < 0.001) and higher discharge mRS scores (4.2 ± 0.7 vs. 2.4 ± 1.3; *p* < 0.001). Table 3 presents the baseline characteristics of the primary cohort, and Supplementary Table S1 presents those of the full cohort aged ≥67 years. Admission nutritional-inflammatory risk was therefore strongly associated with a more severe clinical presentation.

**Table 3:** Baseline characteristics of the primary clinical cohort aged ≥70 years.

Characteristic	Overall (*N* = 300)	Albumin ≥35 g/L (*N* = 213)	Albumin <35 g/L (*N* = 87)	*p*-value	SMD
Age, years	76.8 ± 4.7	76.5 ± 4.6	77.5 ± 4.7	0.121	0.22
Male sex	150 (50.0%)	107 (50.2%)	43 (49.4%)	0.899	0.02
BMI, kg/m^2^	23.9 ± 2.8	24.0 ± 2.8	23.9 ± 2.7	0.710	0.04
Hypertension	262 (87.3%)	189 (88.7%)	73 (83.9%)	0.254	0.14
Diabetes mellitus	165 (55.0%)	117 (54.9%)	48 (55.2%)	0.969	0.01
Atrial fibrillation	27 (9.0%)	13 (6.1%)	14 (16.1%)	0.006	0.32
Coronary artery disease	32 (10.7%)	29 (13.6%)	3 (3.4%)	0.010	0.36
Hyperlipidemia	35 (11.7%)	32 (15.0%)	3 (3.4%)	0.005	0.40
Current/former smoking	25 (8.3%)	20 (9.4%)	5 (5.7%)	0.300	0.14
Alcohol consumption	7 (2.3%)	7 (3.3%)	0 (0.0%)	0.087	0.26
Hemorrhagic stroke	55 (18.3%)	23 (10.8%)	32 (36.8%)	<0.001	0.61
Admission NIHSS	10.5 ± 5.8	8.2 ± 4.8	16.3 ± 3.3	<0.001	1.96
Discharge mRS	2.9 ± 1.4	2.4 ± 1.3	4.2 ± 0.7	<0.001	1.72
Serum albumin, g/L	36.7 ± 2.6	38.0 ± 1.9	33.5 ± 1.0	<0.001	2.96
Prealbumin, mg/L	242.9 ± 40.3	264.9 ± 20.4	189.1 ± 22.1	<0.001	3.56
Admission CRP, mg/L	7.2 ± 3.8	6.6 ± 3.5	8.7 ± 4.3	<0.001	0.54
Cold exposure	38 (12.7%)	31 (14.6%)	7 (8.0%)	0.124	0.21
Mean temperature, °C	17.8 ± 10.8	17.8 ± 10.9	18.0 ± 10.5	0.889	0.02

Data are presented as mean ± standard deviation (SD) or number (percentage), as appropriate. *p*-values were calculated using Student’s t-test for continuous variables and Pearson’s chi-square test or Fisher’s exact test for categorical variables, as appropriate. SMD denotes the absolute standardized mean difference. BMI, body mass index; CRP, C-reactive protein; mRS, modified Rankin Scale; NIHSS, National Institutes of Health Stroke Scale.

#### 3.6.2 Exploratory ceiling-like pattern

**Figure 6:**
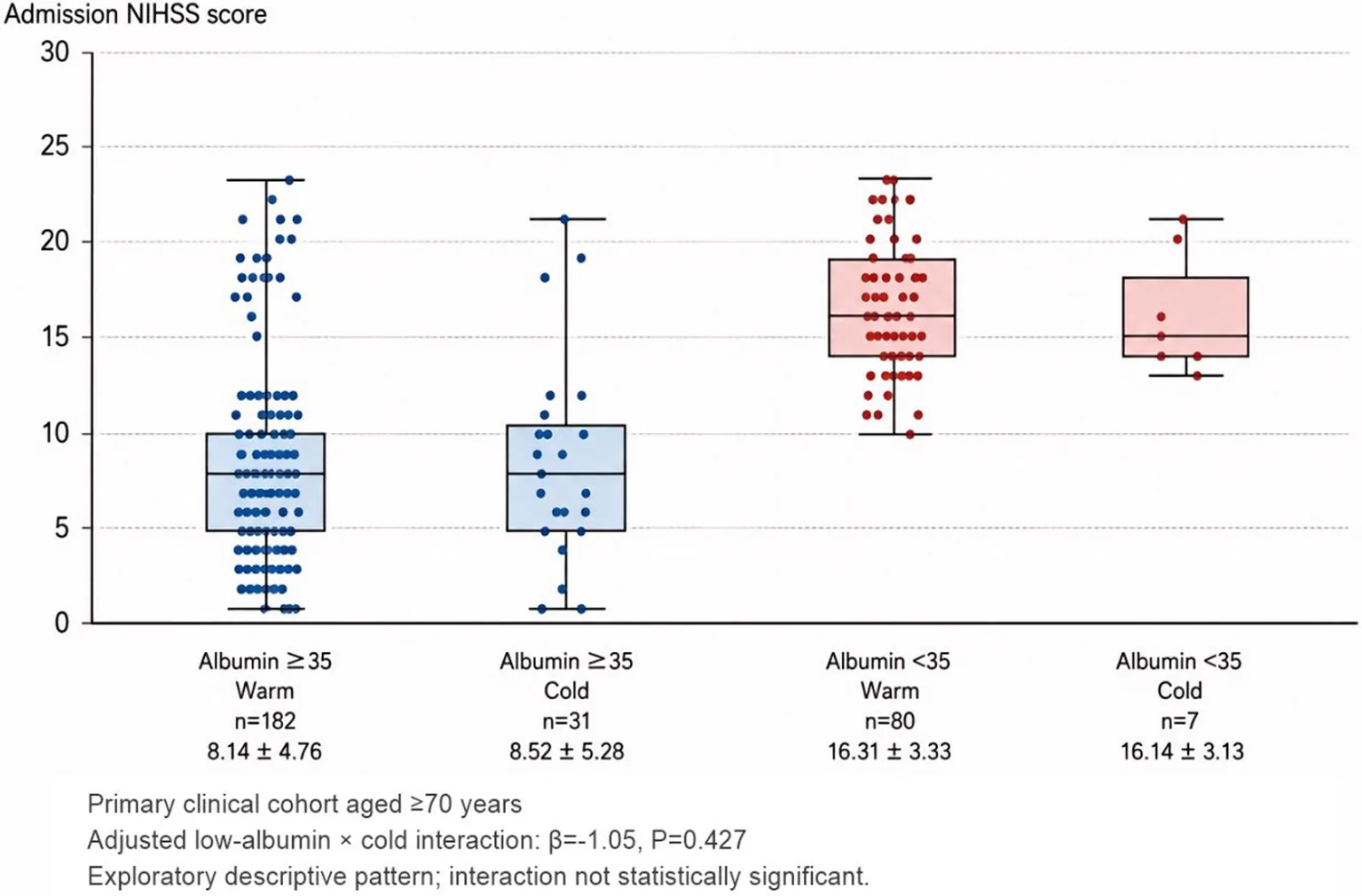
Admission NIHSS by albumin status and cold exposure in the primary clinical cohort aged ≥70 years. Admission National Institutes of Health Stroke Scale (NIHSS) scores are stratified by albumin status and cold exposure in the primary cohort aged ≥70 years. The four groups were normal albumin with warm exposure (albumin ≥35 g/L; *n* = 182), normal albumin with cold exposure (*n* = 31), low albumin with warm exposure (albumin <35 g/L; *n* = 80), and low albumin with cold exposure (*n* = 7). Points represent patients, boxes represent the interquartile range, horizontal lines represent medians, and whiskers show the observed range. The adjusted low-albumin × cold interaction was negative but not statistically significant (*β* = −1.05; *p* = 0.427); the pattern is therefore exploratory. Supplementary Figure S3 shows the corresponding analysis in the full cohort aged ≥67 years.

When stratified by ambient temperature, normally nourished patients aged ≥70 years had admission NIHSS scores of 8.14 ± 4.76 under warm conditions and 8.52 ± 5.28 under cold conditions. Low-albumin patients had persistently high NIHSS scores under both warm and cold conditions (16.31 ± 3.33 vs. 16.14 ± 3.13, respectively). This descriptive pattern was consistent with a ceiling-like hypothesis and is shown in Figure 6, but formal interaction testing was required before drawing mechanistic conclusions.

#### 3.6.3 Multivariable clinical interaction analysis

In the adjusted model restricted to patients aged ≥70 years, low albumin remained associated with a higher NIHSS score (*β* = 5.66, 95% CI, 4.78 to 6.54; *p* < 0.001; Table 4). Cold exposure was not independently associated with NIHSS (*β* = 0.40, 95% CI, −0.74 to 1.53; *p* = 0.495), and the low albumin × cold exposure interaction was negative but not statistically significant (*β* = −1.05, 95% CI, −3.64 to 1.54; *p* = 0.427). Because only 7 patients with low albumin aged ≥70 years were exposed to cold conditions, the interaction estimate was imprecise and should be interpreted as exploratory.

**Table 4:** Multivariable linear regression model for admission NIHSS score in patients aged ≥70 years (*N* = 300).

Predictors	Coefficient (*β*)	95% CI	*p*-value
Primary variables and interaction			
Malnutrition (albumin <35 g/L)	5.66	4.78 to 6.54	< 0.001
Cold exposure (Lag 0–3 temperature < 5 °C)	0.40	−0.74 to 1.53	0.495
Malnutrition × cold exposure	−1.05	−3.64 to 1.54	0.427
Covariates			
Age (years)	0.17	0.10 to 0.25	< 0.001
Sex (Male)	0.19	−0.52 to 0.91	0.601
Body mass index (BMI)	0.19	0.07 to 0.32	0.003
Hypertension	3.54	1.23 to 5.85	0.003
Diabetes mellitus	1.66	0.24 to 3.08	0.023
Atrial fibrillation	3.41	0.81 to 6.01	0.011
Coronary artery disease	0.52	−1.21 to 2.24	0.558
Hyperlipidemia	1.42	−0.30 to 3.14	0.106
Smoking	1.14	−0.15 to 2.44	0.084
Alcohol consumption	2.44	0.10 to 4.78	0.042
Hemorrhagic stroke	9.19	7.77 to 10.60	< 0.001
Admission CRP	0.10	0.00 to 0.19	0.046

The model was restricted to the primary clinical cohort aged ≥70 years and was adjusted for the clinical covariates defined in the Methods. Adjusted *R*^2^ = 0.585. The negative interaction coefficient is reported for transparency and was not statistically significant. BMI, body mass index; CI, confidence interval; CRP, C-reactive protein; NIHSS, National Institutes of Health Stroke Scale.

#### 3.6.4 Sensitivity analyses

The full cohort aged ≥67 years (*N* = 350) yielded similar results. Low albumin was associated with a higher NIHSS score after multivariable adjustment (*β* = 5.64, 95% CI, 4.77 to 6.51; *p* < 0.001). Neither cold exposure (*β* = 0.56, 95% CI, −0.54 to 1.67; *p* = 0.320) nor the low albumin × cold interaction (*β* = −0.89, 95% CI, −3.32 to 1.54; *p* = 0.473) was statistically significant. Supplementary Figure S3 shows the unadjusted four-group pattern in this sensitivity cohort.

Nutritional risk defined by mGNRI <98 was associated with a higher admission NIHSS score (*β* = 6.14, 95% CI, 5.35 to 6.94; *p* < 0.001), whereas the mGNRI × cold interaction was not statistically significant (*β* = −0.87, 95% CI, −2.74 to 1.00; *p* = 0.361). No significant interaction was observed for mean temperature ≥28 °C (albumin status × hot exposure: *β* = −0.54; *p* = 0.542) or the cohort-specific 90th percentile threshold of ≥34.5 °C (*β* = −0.73; *p* = 0.570). Obesity (BMI ≥ 28 kg/m^2^) and its interaction with cold exposure were also not significant. After patients with CRP > 10 mg/L were excluded, low albumin remained associated with higher NIHSS (*β* = 5.79, 95% CI, 4.78 to 6.80; *p* < 0.001), whereas the low albumin × cold interaction remained non-significant. In the functional-outcome sensitivity model, low albumin was associated with a higher discharge mRS score (*β* = 1.26; *p* < 0.001), but the albumin × cold interaction was not significant (*β* = 0.01; *p* = 0.981). Supplementary Table S2 summarizes these analyses.

## 4 Discussion

**Figure 7:**
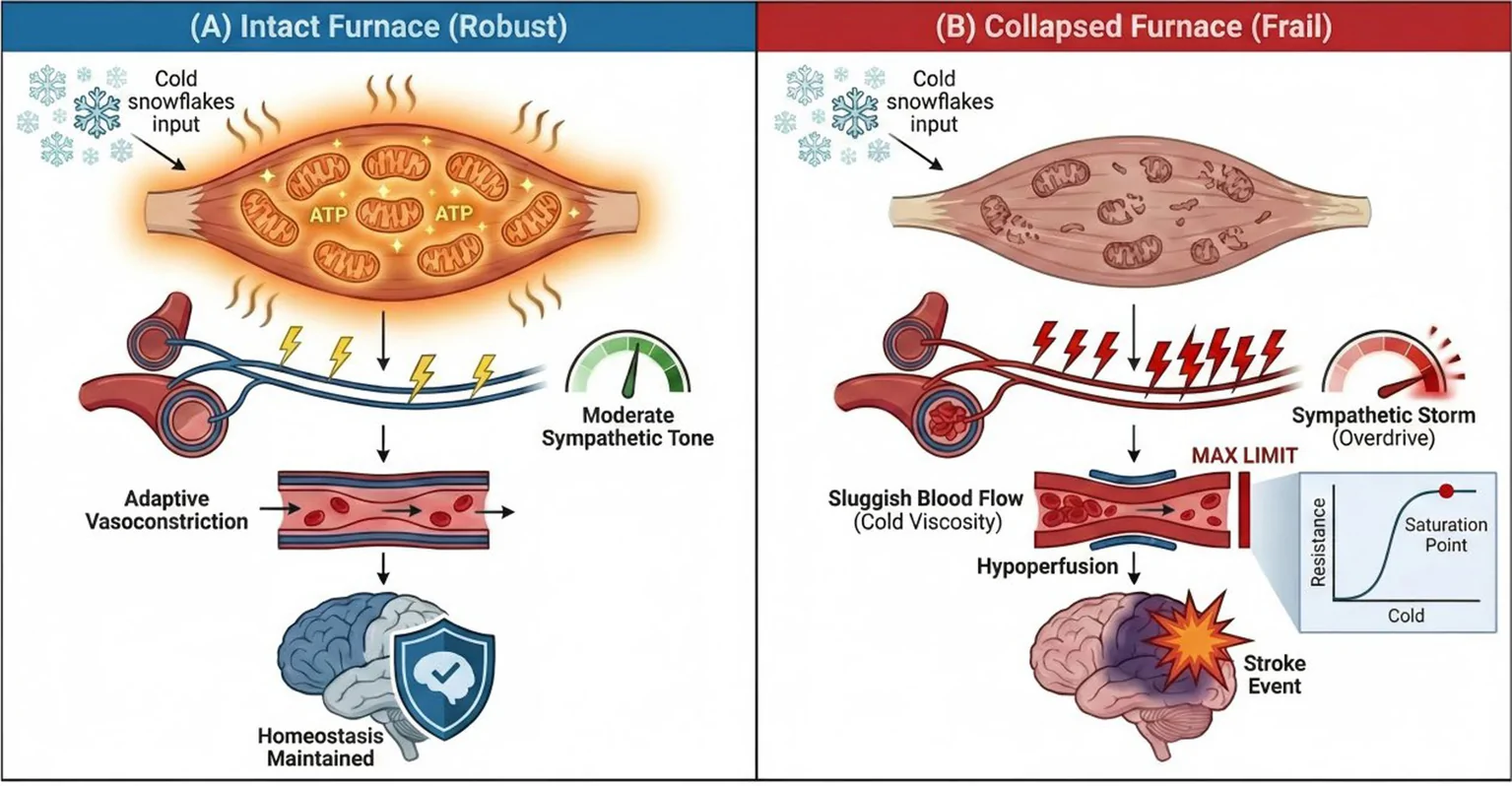
Conceptual schematic of the exploratory “Collapsed Furnace” hypothesis. This schematic illustrates a hypothesis-generating framework linking nutritional depletion, reduced thermogenic reserve, altered vascular response to cold stress, and stroke vulnerability in older adults. **(A)** In robust status, preserved skeletal muscle mass may support heat production and adaptive vasoconstrictive responses during cold exposure, helping maintain circulatory homeostasis. **(B)** In frail or malnourished status, reduced thermogenic reserve may impair cold adaptation and contribute to a ceiling-like vascular response, reduced perfusion, increased stroke vulnerability. The inset illustrates a hypothesized saturation pattern rather than a directly measured physiological threshold. ATP, adenosine triphosphate.

These findings support consideration of both environmental exposure and nutritional reserve when assessing stroke vulnerability in older adults. Across age groups, sodium-attributable burden was most prominent in the younger-old population, whereas cold exposure and nutritional depletion became increasingly relevant in the oldest groups (30–33). The strong association between nutritional risk and stroke severity is compatible with, but does not establish, the sarcopenia-related thermoregulatory framework illustrated in Figure 7 (34–36). Table 1 summarizes the complementary designs, evidentiary roles, and limitations of the three datasets.

### 4.1 Interpreting the risk shift: survivorship bias and selective survival

The relative decline in sodium-attributable stroke burden among adults aged ≥85 years should be interpreted cautiously. It does not imply that dietary sodium becomes physiologically unimportant. Selective survival is one possible explanation: people with greater salt sensitivity or salt-related hypertension may experience cardiovascular events earlier in life (29), leaving an older population that is relatively less susceptible to this specific exposure. At the same time, cold-attributable burden increased at the population level. This pattern is consistent with, but does not prove, greater thermal vulnerability as thermoregulatory capacity declines with age.

### 4.2 The “collapsed furnace” hypothesis: sarcopenia, recovery, and cold adaptation

The “Collapsed Furnace” hypothesis links reduced muscle reserve with a limited capacity to generate and conserve heat. Skeletal muscle is the principal source of shivering thermogenesis in cold-exposed adults and may also contribute to non-shivering thermogenesis through sarcolipin-mediated uncoupling of the sarco/endoplasmic reticulum Ca^2+^-ATPase (SERCA) pump (13, 14). In older adults with preserved reserve, cold exposure elicits coordinated shivering, sympathetic activation, and peripheral vasoconstriction to generate heat and limit heat loss.

Inadequate protein intake may further limit recovery after stroke by reducing amino acid availability for muscle protein synthesis and aggravating the negative protein balance associated with acute illness, immobility, inflammation, and reduced oral intake (37–39). Loss of muscle mass and strength can reduce exercise tolerance, postural control, mobility practice, and participation in rehabilitation, thereby constraining functional recovery and resilience to subsequent physiological stress. Malnutrition and premorbid or post-stroke sarcopenia have been associated with poorer functional outcomes, although estimates vary across populations and definitions (30, 33, 40, 41). Nutritional support combined with rehabilitation may help preserve or restore muscle reserve, but current evidence does not establish that protein supplementation alone improves neurological recovery (37–39).

Reduced muscle reserve may also alter adaptation to ambient cold. Shivering skeletal muscle is a major source of heat production in cold-exposed adults, and muscle may additionally contribute to non-shivering thermogenesis (13, 14, 42). Older adults with reduced muscle mass or impaired muscle quality may therefore generate less metabolic heat and have less substrate reserve during cold exposure (17, 18). This deficit could increase reliance on vasoconstrictive and sympathetic compensation or reduce the capacity to sustain those responses, producing heterogeneous blood-pressure and perfusion patterns in frail individuals. These pathways remain hypothetical in the present study because muscle mass, protein intake, core temperature, autonomic function, and individual cold exposure were not measured.

The CHARLS analysis provided limited physiological context for this framework. Robust participants tended to have higher SBP during cold-season interviews, whereas frail participants did not. However, only 21 participants aged ≥60 years and 4 participants aged ≥70 years were interviewed during cold-season months, and the frailty × cold-season interaction was not statistically significant. These data therefore do not demonstrate autonomic dysregulation and should be interpreted as exploratory.

The proposed “Collapsed Furnace” model should therefore be regarded as a mechanistic hypothesis rather than a demonstrated causal pathway. Frail or sarcopenic individuals may have reduced thermogenic and vasomotor reserve, potentially shifting vulnerability from an acute hypertensive response toward a broader state of limited physiological resilience. Prospective studies that measure muscle mass, muscle strength, dietary intake, core temperature, individual cold exposure, and autonomic markers are required to test this mechanism directly.

### 4.3 Potential ceiling-like pattern: vasomotor unresponsiveness as a hypothesis

The clinical findings require cautious interpretation. Among hospitalized survivors, low albumin was consistently associated with more severe neurological deficits, whereas cold exposure was associated with only a small, non-significant difference in NIHSS. Although the direction of the low albumin × cold interaction resembled the ecological saturation pattern, the clinical interaction was not statistically significant. The findings are therefore hypothesis-generating and do not confirm a ceiling effect. Biological attenuation of thermogenic or vasomotor reserve is one possible explanation, but selection bias, survivor bias, limited power, and exposure misclassification are also plausible.

### 4.4 Implications for prevention

Clinically, these findings support attention to nutritional risk in older patients with stroke and to environmental protection during cold periods. The ceiling-like pattern should not be interpreted as evidence that cold exposure is harmless in malnourished individuals. Nutritional assessment and support, infection prevention, adequate heating, and individualized thermal protection should instead be considered complementary measures for frail older adults with limited physiological reserve (37–40, 43, 44).

### 4.5 Limitations

Several limitations should be considered. First, the ecological GBD analysis cannot support causal inference at the individual level. Second, serum albumin was measured after stroke onset and is not a definitive indicator of pre-stroke nutritional status. As a negative acute-phase reactant, albumin may reflect chronic nutritional depletion, acute inflammation, or both. Adjustment for admission CRP, the CRP-restricted analysis, and the mGNRI analysis reduced but did not eliminate this concern. Third, the clinical cohort included hospitalized survivors and did not capture pre-hospital deaths or fatal cases not admitted to our center. The small cold-related difference in NIHSS among patients with low albumin should therefore not be interpreted as absence of harm. Fourth, the clinical interaction analysis had limited power because only 7 cold-exposed patients aged ≥70 years had low albumin. Fifth, the meteorological exposure represented area-level outdoor temperature and did not capture indoor heating, clothing, outdoor or physical activity, or personal thermal protection. Sixth, the retrospective dataset lacked pre-stroke albumin, core temperature, grip strength, muscle mass, detailed infection data, liver and renal function, and mortality. Supplementary Table S3 lists these unavailable variables and the mitigation analyses. Finally, the single-center Shanghai cohort may not generalize to colder regions or settings with different heating infrastructure. Future prospective studies should include pre-stroke nutritional assessment using the Geriatric Nutritional Risk Index or Global Leadership Initiative on Malnutrition (GLIM) criteria, direct muscle measurements, core temperature, individual exposure, physical activity, infection status, and mortality outcomes.

## 5 Conclusion

Using a multilevel observational approach, we found that nutritional depletion was strongly associated with stroke severity in older adults, whereas its potential role in modifying cold-related vulnerability remains uncertain. The ecological interaction and the direction of the clinical findings were compatible with an exploratory ceiling-like pattern, but the clinical interaction was not statistically significant and the “Collapsed Furnace” mechanism remains hypothetical. Prospective multicenter studies with pre-stroke nutritional assessment, direct muscle and temperature measurements, individual exposure data, and mortality outcomes are needed to evaluate this framework.

## Data Availability

The GBD data used in this study are publicly available from the Institute for Health Metrics and Evaluation (IHME), and the CHARLS data are available through the China Health and Retirement Longitudinal Study data repository. De-identified clinical data are available from the corresponding author upon reasonable request, subject to institutional approval and applicable privacy restrictions.
