# Supplementary material for: Nutritional risk, cold exposure, and stroke severity in older adults: an exploratory analysis of the “collapsed furnace” hypothesis
Source: Front Public Health. 2026 Aug 26;14:1838018. doi: 10.3389/fpubh.2026.1838018 (PMC13553406; doi:10.3389/fpubh.2026.1838018)
Supplement: Supplementary file 1 [file Supplementary_file_1.docx]

**Supplementary Materials**

**Supplementary Methods. Exposure Definition and Interaction Models**

**Cold exposure definition**

Cold exposure was defined as Lag 0–3 average daily mean temperature <5 °C. Lag 0–3 temperature was calculated as the mean temperature on the day of stroke onset and the preceding three days using data from a representative regional meteorological station.

**Heat exposure definition**

Hot exposure was evaluated using two thresholds: Lag 0–3 average daily mean temperature ≥28 °C and the cohort-specific 90th percentile threshold of ≥34.5 °C.

**Clinical interaction model**

The primary clinical model used the admission National Institutes of Health Stroke Scale (NIHSS) score as a continuous dependent variable:

NIHSS = β0 + β1 nutritional risk + β2 cold exposure + β3 nutritional risk × cold exposure + covariates + ε

The primary clinical cohort was restricted to patients aged ≥70 years. The full cohort aged ≥67 years was retained as a sensitivity analysis.

**Covariates**

Clinical models were adjusted for age, sex, body mass index (BMI), hypertension, diabetes mellitus, atrial fibrillation, coronary artery disease, hyperlipidemia, smoking, alcohol consumption, stroke type, and admission C-reactive protein (CRP).

Modified Geriatric Nutritional Risk Index

As a BMI-adjusted nutritional sensitivity analysis, the modified Geriatric Nutritional Risk Index (mGNRI) was calculated as:

mGNRI = 1.489 × serum albumin (g/L) + 41.7 × min(BMI/22, 1)

Nutritional risk was defined as mGNRI <98.

**Supplementary Table S1. Baseline characteristics of the full clinical cohort aged ≥67 years**

| **Variable** | **Total cohort (N=350)** | **Albumin ≥35 g/L (n=250)** | **Albumin <35 g/L (n=100)** | **P value** |
| --- | --- | --- | --- | --- |
| **Demographics** |  |  |  |  |
| Age, years | 75.6 ± 5.3 | 75.3 ± 5.2 | 76.3 ± 5.4 | 0.116 |
| Male sex | 176 (50.3%) | 125 (50.0%) | 51 (51.0%) | 0.866 |
| BMI, kg/m² | 24.0 ± 2.8 | 24.1 ± 2.8 | 23.8 ± 2.7 | 0.289 |
| **Vascular comorbidities and risk factors** |  |  |  |  |
| Hypertension | 305 (87.1%) | 224 (89.6%) | 81 (81.0%) | 0.030 |
| Diabetes mellitus | 195 (55.7%) | 140 (56.0%) | 55 (55.0%) | 0.865 |
| Atrial fibrillation | 33 (9.4%) | 14 (5.6%) | 19 (19.0%) | <0.001 |
| Coronary artery disease | 38 (10.9%) | 34 (13.6%) | 4 (4.0%) | 0.008 |
| Hyperlipidemia | 41 (11.7%) | 37 (14.8%) | 4 (4.0%) | 0.003 |
| Current/former smoking | 33 (9.4%) | 27 (10.8%) | 6 (6.0%) | 0.165 |
| Alcohol consumption | 13 (3.7%) | 11 (4.4%) | 2 (2.0%) | 0.363 |
| **Stroke characteristics** |  |  |  |  |
| Stroke type |  |  |  | <0.001 |
| — Ischemic stroke | 287 (82.0%) | 224 (89.6%) | 63 (63.0%) |  |
| — Hemorrhagic stroke | 63 (18.0%) | 26 (10.4%) | 37 (37.0%) |  |
| Onset-to-admission time, h, median [IQR] | 24 [24–24] | 24 [24–24] | 24 [24–24] | 0.165 |
| Admission NIHSS score | 10.3 ± 6.0 | 7.9 ± 4.9 | 16.3 ± 3.8 | <0.001 |

Abbreviations: BMI, body mass index; IQR, interquartile range; NIHSS, National Institutes of Health Stroke Scale.

**Supplementary Table S2. Summary of clinical sensitivity analyses**

| **Analysis** | **Sample** | **Exposure** | **Main effect** | **Interaction term** | **Conclusion** |
| --- | --- | --- | --- | --- | --- |
| Primary analysis | ≥70 years, N=300 | Albumin <35 g/L | β = 5.66, P < 0.001 | Albumin <35 × cold: β = -1.05, P = 0.427 | Nutritional risk was strongly associated with NIHSS; interaction was not significant. |
| Sensitivity 1 | ≥67 years, N=350 | Albumin <35 g/L | β = 5.64, P < 0.001 | Albumin <35 × cold: β = -0.89, P = 0.473 | Directionally consistent with the primary analysis. |
| Sensitivity 2 | Full cohort | mGNRI <98 | β = 6.14, P < 0.001 | mGNRI <98 × cold: β = -0.87, P = 0.361 | Alternative nutritional index gave consistent nutritional-risk association. |
| Sensitivity 3 | CRP ≤10 mg/L, N=296 | Albumin <35 g/L | β = 5.79, P < 0.001 | Albumin <35 × cold: β = -1.71, P = 0.365 | Findings persisted after excluding marked inflammation. |
| Sensitivity 4 | Full cohort | Hot exposure ≥28 °C | Not primary estimand | Albumin <35 × hot: β = -0.54, P = 0.542 | Heat interaction was not significant. |
| Sensitivity 5 | Full cohort | Extreme hot exposure ≥34.5 °C | Not primary estimand | Albumin <35 × extreme hot: β = -0.73, P = 0.570 | Extreme-heat interaction was not significant. |
| Sensitivity 6 | Full cohort | mRS outcome | Albumin <35: β = 1.26, P < 0.001 | Albumin <35 × cold: β = 0.01, P = 0.981 | Nutritional risk was associated with functional outcome; interaction was not significant. |
| Sensitivity 7 | Full cohort | Obesity, BMI ≥28 kg/m² | Obesity: β = 0.92, P = 0.123 | Obesity × cold: β = -0.84, P = 0.606 | Obesity did not show a significant cold interaction. |

Note: The primary interaction analysis was imprecise because only 7 patients with low albumin aged ≥70 years were exposed to cold conditions. Abbreviations: BMI, body mass index; CRP, C-reactive protein; mGNRI, modified Geriatric Nutritional Risk Index; mRS, modified Rankin Scale; NIHSS, National Institutes of Health Stroke Scale.

**Supplementary Table S3. Reviewer-requested variables unavailable in the retrospective dataset**

| **Reviewer-requested variable** | **Availability** | **Handling in revised analysis/manuscript** |
| --- | --- | --- |
| Pre-stroke serum albumin | Unavailable | Admission albumin was reframed as a nutritional-inflammatory risk marker and this limitation was stated. |
| Standard Geriatric Nutritional Risk Index (GNRI) components | Incomplete | A BMI-adjusted mGNRI was used as sensitivity analysis. |
| Grip strength or muscle mass in clinical cohort | Unavailable | CHARLS was retained only as exploratory physiological context. |
| Core body temperature | Unavailable | Listed as a variable for future prospective studies. |
| Individual indoor temperature | Unavailable | Regional meteorological Lag 0–3 temperature was used; exposure misclassification was acknowledged. |
| Outdoor activity, clothing, and heating | Unavailable | Acknowledged as unmeasured exposure determinants. |
| Detailed influenza, pneumonia, or infection data | Unavailable | Admission CRP adjustment and CRP ≤10 mg/L sensitivity analysis were used. |
| Liver and renal dysfunction | Unavailable | Residual confounding was acknowledged. |
| Mortality and mRS=6 | Unavailable | Hospitalized-survivor selection bias was emphasized; discharge mRS sensitivity was added. |

Abbreviations: BMI, body mass index; CHARLS, China Health and Retirement Longitudinal Study; CRP, C-reactive protein; GNRI, Geriatric Nutritional Risk Index; mGNRI, modified Geriatric Nutritional Risk Index; mRS, modified Rankin Scale.

**Supplementary Figure S1. Analysis framework and evidentiary role**

Analysis framework showing the evidentiary role of each dataset and analysis layer. The GBD 2023 analysis, restricted to 1990–2021 estimates, was used to describe population-level age-risk transition and ecological interaction between malnutrition prevalence and low-temperature burden. CHARLS 2015 was used only as exploratory physiological context for seasonal SBP patterns because cold-season interviews were sparse. The retrospective clinical cohort provided individual-level stroke severity analyses, including the primary age ≥70 cohort, the full age ≥67 sensitivity cohort, mGNRI, CRP-restricted, heat, obesity, and mRS sensitivity analyses. Interpretation safeguards explicitly acknowledge that the clinical interaction was not statistically significant, the low-albumin cold-exposed subgroup was small (n=7), and several reviewer-requested variables were unavailable. This framework is intended to clarify triangulation of evidence and should not be interpreted as causal or confirmatory mechanistic proof. Abbreviations: GBD, Global Burden of Disease; CHARLS, China Health and Retirement Longitudinal Study; SBP, systolic blood pressure; NIHSS, National Institutes of Health Stroke Scale; mGNRI, modified Geriatric Nutritional Risk Index; CRP, C-reactive protein; mRS, modified Rankin Scale.

**Supplementary Figure S2. Flowchart of participant selection from the CHARLS 2015 wave**

The flowchart illustrates the sequential derivation of the CHARLS 2015 analytic samples used for the exploratory physiological context analysis. From the initial CHARLS 2015 cohort (N=21,095), participants were retained if they had complete data on age, sex, interview month, three systolic blood pressure measurements, grip strength, height, and weight (N=15,373). The age ≥60 years analytic sample included N=7,633 participants, among whom only 21 had cold-season interviews. The age ≥70 years subset included N=2,739 participants, among whom only 4 had cold-season interviews. Because cold-season interviews were sparse, the CHARLS analysis was interpreted as exploratory physiological context rather than confirmatory mechanistic evidence. CHARLS, China Health and Retirement Longitudinal Study; SBP, systolic blood pressure.

**Supplementary Figure S3. Admission NIHSS by albumin status and cold exposure in the full cohort aged ≥67 years**

Mean admission National Institutes of Health Stroke Scale (NIHSS) scores (± standard deviation [SD]) are stratified by admission albumin status and cold exposure in the full cohort aged ≥67 years (N=350). This figure is the sensitivity counterpart to the primary analysis in patients aged ≥70 years shown in main-text Figure 6. The adjusted low-albumin × cold interaction was negative but not statistically significant (β = −0.89; p = 0.473).
